# Clinicopathological characteristics of gastric neuroendocrine neoplasms: A comprehensive analysis

**DOI:** 10.1002/cam4.7011

**Published:** 2024-03-08

**Authors:** Mengli Zi, Yubo Ma, Jinxia Chen, Chuhong Pang, Xiao Li, Li Yuan, Zhuo Liu, Pengfei Yu

**Affiliations:** ^1^ Postgraduate training base Alliance of Wenzhou Medical University (Zhejiang Cancer Hospital) Hangzhou Zhejiang China; ^2^ Department of Gastric surgery, Zhejiang Cancer Hospital Hangzhou Institute of Medicine (HIM), Chinese Academy of Sciences Hangzhou Zhejiang China; ^3^ Zhejiang Provincial Research Center for Upper Gastrointestinal Tract Cancer Zhejiang Cancer Hospital Hangzhou China; ^4^ Zhejiang Key Lab of Prevention, Diagnosis and Therapy of Upper Gastrointestinal Cancer Zhejiang Cancer Hospital Hangzhou China; ^5^ The Second Clinical Medical College of Zhejiang Chinese Medical University Hangzhou Zhejiang China; ^6^ Department of Colorectum surgery Zhejiang Cancer Hospital, Hangzhou Institute of Medicine (HIM), Chinese Academy of Sciences Hangzhou Zhejiang China

**Keywords:** gastric mixed neuroendocrine–non‐neuroendocrine neoplasms, gastric neuroendocrine carcinomas, gastric neuroendocrine neoplasms, gastric neuroendocrine tumors, pathological feature, prognosis

## Abstract

**Objective:**

This study aimed to explore the clinicopathological characteristics and prognostic implications of gastric neuroendocrine neoplasms (g‐NENs).

**Methods:**

A retrospective enrollment of 142 patients diagnosed with g‐NENs was conducted at Zhejiang Cancer Hospital between January 1, 2007 and December 31, 2021. The study compared essential clinicopathological features and survival rates. Additionally, the prognosis of gastric neuroendocrine carcinomas/mixed neuroendocrine–non‐neuroendocrine neoplasms (g‐NEC/MiNEN) were contrasted with those of gastric adenocarcinoma (GAC) and signet ring cell carcinoma (SRCC).

**Results:**

The study comprised a total of 142 g‐NENs cases, with a male‐to‐female ratio of approximately 2:1. The 5‐year survival rates for g‐NEC and g‐MiNEN were 26.7% and 35.2%, respectively. Corresponding 5‐year survival rates for G1 and G2 were observed at 100% and 80.0%, respectively. g‐NEC/MiNEN showed a significantly worse prognosis compared to g‐NET (*p* < 0.001). g‐NEC/MiNEN exhibited a poor prognosis compared to GAC (*p* < 0.001), and within poorly differentiated GAC, g‐NEC/MiNEN demonstrated a worse prognosis (*p* = 0.007). Additionally, patients receiving postoperative adjuvant therapy exhibited notably prolonged overall survival (OS) in the case of g‐NEC/MiNEN (*p* = 0.010).

**Conclusion:**

In short, the prognosis of g‐NEC/MiNEN was worse than that of g‐NET, GAC and poorly differentiated GAC, but this group benefit from postoperative adjuvant therapy.

## Graphical abstract

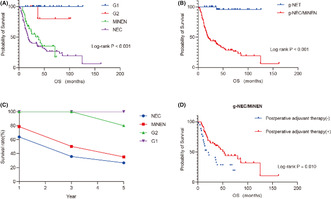

## 1 INTRODUCTION

Neuroendocrine neoplasms (NENs) represent a rare category of neoplasms, accounting for just 0.5% of all malignant neoplasms. They can manifest in various body parts, with the stomach, pancreas, and lungs being the most common sites.^1^, ^2^, ^3^, ^4^ Gastric neuroendocrine neoplasms (g‐NENs) are even rarer, constituting approximately 7% of all gastrointestinal neuroendocrine tumors,^5^ and a mere 0.1%–0.6% of all gastric tumors.^6^ The incidence of g‐NENs has markedly increased in recent years, likely attributed to heightened clinician awareness, improved diagnostic techniques, and widespread utilization of upper gastrointestinal endoscopy.^7^

As per the latest 2019 edition of the WHO Classification of Digestive System Tumors, NENs are classified into three distinct categories: well‐differentiated neuroendocrine tumors (NETs), poorly differentiated neuroendocrine carcinomas (NECs), and mixed neuroendocrine‐non‐neuroendocrine neoplasms (MiNEN).^8^ The NET classification was further refined into G1, G2, and G3 based on grade, Mitotic rate, and Ki‐67 index. Meanwhile, NEC is categorized into large‐cell type (LCNEC) and small‐cell type (SCNEC) based on cellular morphology,^8^ as delineated in Table 1. The majority of NENs fall under the well‐differentiated neuroendocrine tumors (NETs) category, exhibiting indolent biological behavior and longer survival times.^5^, ^9^ By contrast, NECs display a higher malignant potential, poorer prognosis, and shorter survival times.^10^ g‐NEC and g‐MiNEN, as rare subtypes of gastric cancer, constitute just 0.6% of all gastric malignancies.^11^, ^12^, ^13^ The limited understanding of g‐NENs is due to their low incidence and high heterogeneity.

**TABLE 1:** Clinicopathological characteristics of 142 cases of gastric neuroendocrine neoplasms.

Variable	g‐NEN (142) *N* (%)	G1 (23) *N* (%)	G2 (8) *N* (%)	MiNEN (28) *N* (%)	NEC (83) *N* (%)
Age (year)	63.00 (55.75, 69.00)	50.00 (47.00, 56.00)	54.50 (50.00, 58.00)	68.00 (58.50, 70.00)	66.00 (60.00, 70.00)
Tumor size (cm)	4.00 (2.50, 6.00)	0.70 (0.40, 0.80)	1.75 (0.85, 2.50)	4.50 (3.00, 6.00)	5.00 (3.50, 7.00)
LNR (%)	11.11 (0.00, 21.74)	0.00 (0.00,0.00)	0.00 (0.00, 44.79)	15.56 (2.63, 26.92)	11.11 (0.00, 20.80)
Ki‐67 (%)	60.00 (30.00, 80.00)	2.00 (1.00, 2.00)	5.00 (3.00, 9.25)	80.00 (60.00, 80.00)	70.00 (60.00, 80.00)
BMI	21.78 (20.16, 24.57)	21.30 (20.81, 24.96)	23.24 (21.31, 24.11)	23.42 (20.78, 26.11)	21.19 (19.42, 24.12)
Gender					
Male	97 (68.3)	6 (26.1)	2 (25.0)	21 (75.0)	68 (81.9)
Female	45 (31.7)	17 (73.9)	6 (75.0)	7 (25.0)	15 (18.1)
Smoking history					
No	84 (59.2)	21 (91.3)	6 (75.0)	17 (60.7)	40 (48.2)
Yes	58 (40.8)	2 (8.7)	2 (25.0)	11 (39.3)	43 (51.8)
Drinking history					
No	93 (65.5)	19 (82.6)	5 (62.5)	19 (67.9)	50 (60.2)
Yes	49 (34.5)	4 (17.4)	3 (37.5)	9 (32.1)	33 (39.8)
Family history					
No	103 (72.5)	16 (69.6)	6 (75.0)	20 (71.4)	61 (73.5)
Yes	39 (27.5)	7 (30.4)	2 (25.0)	8 (28.6)	22 (26.5)
Weight loss					
No	93 (65.5)	22 (95.7)	6 (75.0)	19 (67.9)	46 (55.4)
Yes	49 (34.5)	1 (4.3)	2 (25.0)	9 (32.1)	37 (44.6)
BMI					
<18.5	11 (7.7)	–	1 (12.5)	–	10 (12.0)
18.5–24.0	88 (62.0)	16 (69.6)	5 (62.5)	17 (60.7)	50 (60.2)
>24.0	42 (29.6)	7 (30.4)	2 (25.0)	11 (39.3)	22 (26.5)
Unknown	1 (0.7)	–	–	–	1 (1.2)
Symptom					
Abdominal distension and pain	96 (67.6)	11 (47.8)	4 (50.0)	17 (60.7)	64 (77.1)
Obstruction of swallowing	14 (9.9)	–	–	5 (17.9)	9 (10.8)
Hematemesis, black stool	8 (5.6)	–	2 (25.0)	2 (7.1)	4 (4.8)
Fatigue	2 (1.4)	1 (4.3)	–	–	1 (1.2)
No	22 (15.5)	11 (47.8)	2 (25.0)	4 (14.3)	5 (6.0)
Recurrence or metastasis					
No	85 (59.9)	20 (87.0)	4 (50.0)	18 (64.3)	43 (51.8)
Yes	57 (40.1)	3 (13.0)	4 (50.0)	10 (35.7)	40 (48.2)
Distant metastasis in initial diagnosis					
No	107 (75.4)	23 (100.0)	6 (75.0)	23 (82.1)	55 (66.3)
Yes	35 (24.6)	–	2 (25.0)	5 (17.9)	28 (33.7)
Recurrent or metastatic sites					
Locality	7 (12.3)	3 (100.0)	2 (50.0)	–	2 (5.0)
Distant lymph node	17 (29.8)	–	–	6 (60.0)	11 (27.5)
Liver	27 (47.4)	–	2 (50.0)	4 (40.0)	21 (52.5)
Lung	2 (3.5)	–	–	–	2 (5.0)
Bone	1 (1.8)	–	–	–	1 (2.5)
Brain	1 (1.8)	–	–	–	1 (2.5)
Vermiform appendix	1 (1.8)	–	–	–	1 (2.5)
Adrenal gland	1 (1.8)	–	–	–	1 (2.5)
Neoadjuvant therapy					
No	97 (84.3)	23 (100.0)	7 (100.0)	22 (81.5)	45 (77.6)
Yes	18 (15.7)	–	–	5 (18.5)	13 (22.4)
Postoperative adjuvant therapy					
No	61 (53.0)	23 (100.0)	6 (85.7)	9 (33.3)	23 (39.7)
Yes	54 (47.0)	–	1 (14.3)	18 (66.7)	35 (60.3)
Survival state					
Alive	64 (45.1)	23 (100.0)	7 (87.5)	11 (39.3)	23 (27.7)
Death	78 (54.9)	–	1 (12.5)	17 (60.7)	60 (72.3)
Nerve invasion					
No	56 (39.4)	9 (39.1)	5 (62.5)	12 (42.9)	30 (36.1)
Yes	43 (30.3)	–	–	15 (53.6)	28 (33.7)
Unknown	43 (30.3)	14 (60.9)	3 (37.5)	1 (3.6)	25 (30.1)
Vascular tumor thrombus					
No	40 (28.2)	8 (34.8)	2 (25.0)	8 (28.6)	22 (26.5)
Yes	59 (41.5)	1 (4.3)	3 (37.5)	19 (67.9)	36 (43.4)
Unknown	43 (30.3)	14 (60.9)	3 (37.5)	1 (3.6)	25 (30.1)
LNR(%)					
≤11.65	51 (35.9)	9 (39.1)	3 (37.5)	9 (32.1)	30 (36.1)
>11.65	48 (33.8)	–	2 (25.0)	18 (64.3)	28 (33.7)
Unknown	43 (30.3)	14 (60.9)	3 (37.5)	1 (3.6)	25 (30.1)
Depth of infiltration					
Mucous membrane	12 (8.5)	10 (43.5)	1 (12.5)	–	1 (1.2)
Submucosa	20 (14.1)	10 (43.5)	5 (62.5)	1 (3.6)	4 (4.8)
Muscular layer	12 (8.5)	1 (4.3)	1 (12.5)	2 (7.1)	8 (9.6)
Subserous membrane	6 (4.2)	–	–	2 (7.1)	4 (4.8)
Serous membrane and beyond	64 (45.1)	–	1 (12.5)	22 (78.6)	41 (49.4)
Unknown	28 (19.7)	2 (8.7)	–	1 (3.6)	25 (30.1)
Tumor location					
Upper 1/3 of the stomach	59 (41.5)	5 (21.7)	–	12 (42.9)	42 (50.6)
Middle 1/3 of the stomach	51 (35.9)	17 (73.9)	5 (62.5)	8 (28.6)	21 (25.3)
Lower 1/3 of the stomach	32 (22.5)	1 (4.3)	3 (37.5)	8 (28.6)	20 (24.1)
Range of surgery					
Whole stomach	42 (29.6)	8 (34.8)	3 (42.9)	20 (74.1)	42 (72.4)
Partial stomach	73 (51.4)	15 (65.2)	4 (57.1)	7 (25.9)	16 (27.6)
Objective of surgery					
Radical	112 (97.4)	23 (100.0)	6 (85.7)	26 (96.3)	57 (98.3)
Palliative	3 (2.6)	–	1 (14.3)	1 (3.7)	1 (1.7)
Method of surgery					
Open	81 (70.4)	4 (17.4)	3 (42.9)	23 (85.2)	51 (87.9)
Laparoscope	18 (15.7)	5 (21.7)	2 (28.6)	4 (14.8)	7 (12.1)
ESD	16 (13.9)	14 (60.9)	2 (28.6)	–	–
T stage					
1	14 (9.9)	8 (34.8)	1 (12.5)	1 (3.6)	4 (4.8)
2	16 (11.3)	2 (8.7)	4 (50.0)	2 (7.1)	8 (9.6)
3	6 (4.2)	–	–	2 (7.1)	4 (4.8)
4	64 (45.1)	–	1 (12.5)	22 (78.6)	41 (49.4)
Unknown	42 (29.6)	13 (56.5)	2 (25.0)	1 (3.6)	26 (31.3)
N stage					
0	49 (34.5)	–	5 (62.5)	6 (21.4)	16 (19.3)
1	16 (11.3)	22 (95.7)	2 (25.0)	2 (7.1)	11 (13.3)
2	27 (19.0)	1 (4.3)	–	10 (35.7)	17 (20.5)
3	22 (15.5)	–	–	9 (32.1)	13 (15.7)
Unknown	28 (19.7)	–	1 (12.5)	1 (3.6)	26 (31.3)
M stage					
0	114 (80.3)	23 (100.0)	6 (75.0)	26 (92.9)	59 (71.1)
1	28 (19.7)	–	2 (25.0)	2 (7.1)	24 (28.9)
TNM stage					
I	18 (12.7)	8 (34.8)	1 (12.5)	3 (10.7)	6 (7.2)
II	21 (14.8)	1 (4.3)	3 (37.5)	3 (10.7)	14 (16.9)
III	54 (38.0)	1 (4.3)	1 (12.5)	20 (71.4)	32 (38.6)
IV	28 (19.7)	–	2 (25.0)	2 (7.1)	24 (28.9)
Unknown	21 (14.8)	13 (56.5)	1 (12.5)	–	7 (8.4)
HER2					
Negative	86 (60.6)	3 (13.0)	2 (25.0)	18 (64.3)	63 (75.9)
Positive	9 (6.3)	–	–	8 (28.6)	1 (1.2)
Unknown	47 (33.1)	20 (87.0)	6 (75.0)	2 (7.1)	19 (22.9)
CgA					
Negative	30 (21.1)	–	–	10 (35.7)	20 (24.1)
Positive	107 (75.4)	21 (91.3)	8 (100.0)	18 (64.3)	60 (72.3)
Unknown	5 (3.5)	2 (8.7)	–	–	3 (3.6)
Sy					
Negative	–	–	–	–	–
Positive	137 (96.5)	21 (91.3)	8 (100.0)	27 (96.4)	81 (97.6)
Unknown	5 (3.5)	2 (8.7)	–	1 (3.6)	2 (2.4)
CD56					
Negative	23 (16.2)	1 (4.3)	1 (12.5)	6 (21.4)	15 (18.1)
Positive	97 (68.3)	20 (87.0)	6 (75.0)	19 (67.9)	52 (62.7)
Unknown	22 (15.5)	2 (8.7)	1 (12.5)	3 (10.7)	16 (19.3)

The prognosis of g‐NETs and g‐NEC/MiNENs diverges significantly due to differences in differentiation degree and biological behavior. While g‐NETs manifest indolent growth patterns and tend to be benign, g‐NECs are highly malignant, invasive, and associated with dismal prognoses. Various types of g‐NENs present distinct clinical attributes, treatment approaches, pathological traits, and prognostic outcomes. Therefore, it is imperative to study them separately based on their pathological subtypes. In this study, we conducted a retrospective analysis of 142 cases of g‐NENs from Zhejiang Cancer Hospital to investigate their clinicopathological features and prognostic factors. Furthermore, we compared the clinicopathological characteristics and outcomes of g‐NEC/MiNEN, gastric adenocarcinoma (GAC), and gastric signet ring cell carcinoma (SRCC), with the objective of deepening our comprehension of g‐NENs.

## 2 MATERIALS AND METHODS

### 2.1 Patient samples

**FIGURE 1:**
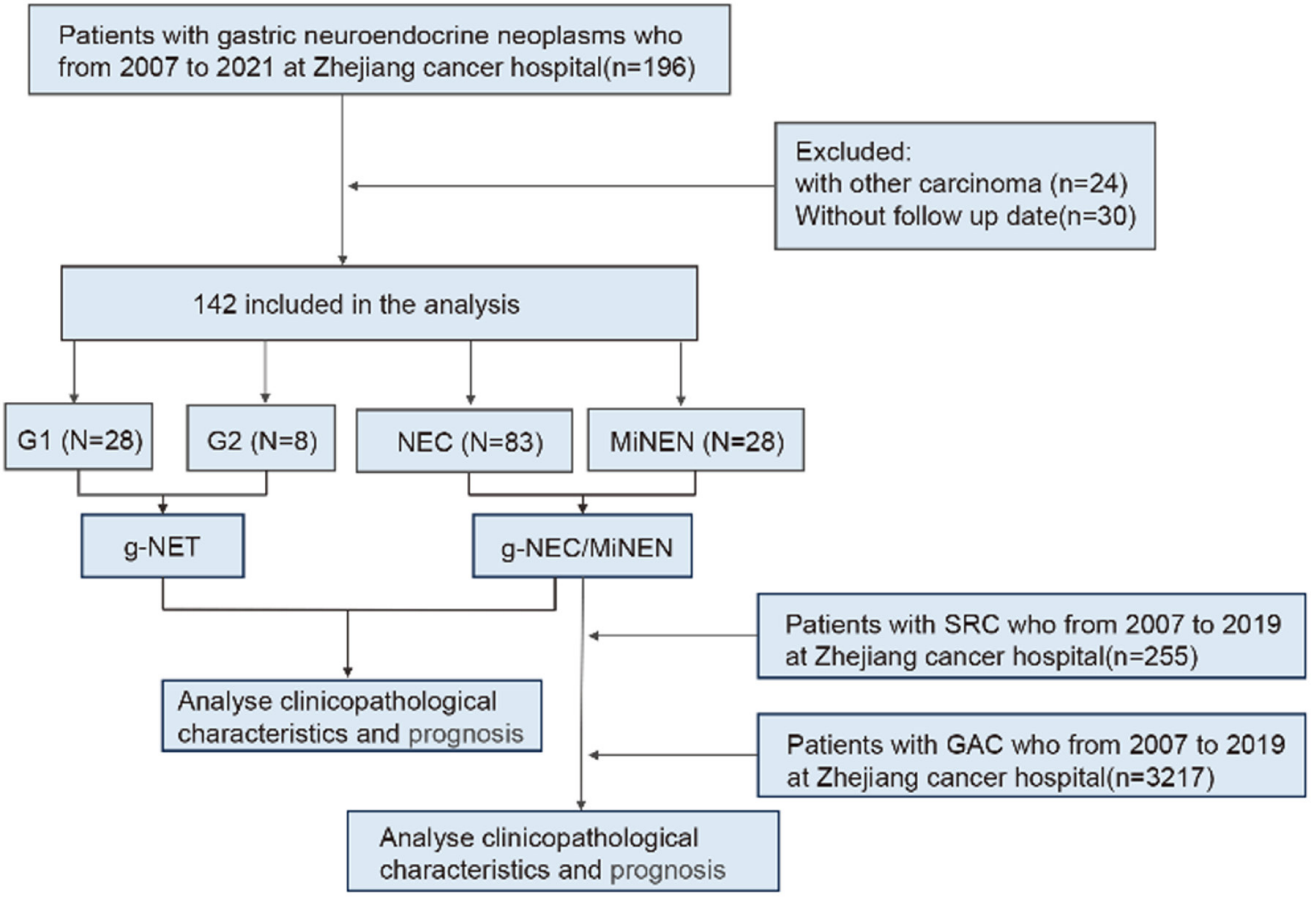
Flowchart for the selection process of study objects.

In this study, we included patients diagnosed with primary gastric neuroendocrine neoplasms and admitted to Zhejiang Cancer Hospital between January 1, 2007 and December 31, 2021. Inclusion criteria were as follows: (1) Pathological examination of surgical specimens or gastroscopic biopsy specimens confirming primary gastric neuroendocrine neoplasms; (2) Availability of relatively comprehensive clinical and pathological data. Exclusion criteria were as follows: (1) Presence of concomitant malignant tumors; (2) Nonprimary tumors that have metastasized to the stomach from other sites; (3) Patients with severe cardiopulmonary, liver, or kidney dysfunction; (4) Absence of follow‐up data. The selection process is visually presented in Figure 1.

### 2.2 Pathology methods

According to the 2019 WHO Classification criteria for Digestive System tumors (5th edition), gastric neuroendocrine neoplasms were meticulously classified by two senior pathologists and further subdivided into G1, G2, G3, NEC, and MiNEN. The diagnosis criteria for those 28 cases of MiNEN are detailed in Table S1. All pathological stages were aligned with AJCC 8th criteria. Patient historical data, encompassing demographic characteristics and clinicopathological characteristics, were systematically extracted from inpatient records. Survival data were procured through telephone follow‐ups or medical records, with the final follow‐up conducted on January 1st, 2023. The overall survival (OS) was defined as the duration from initial surgery or first diagnosis to death or last follow‐up date.

### 2.3 Immunohistochemistry

HER2: Tissue sections were baked at 60°C for 60 min and then placed in ventana BenchMark Ultra for staining. The procedure is as follows: EZ prep dewaxing, ULTRA CC1 antigen repair for 36 min, HER2 antibody incubation at 37°C for 32 min, ultraView Universal DAB Detection Kit detection system color rendering, hematoxylin II nuclear restaining. Bluing reagent returns blue. For other antibodies: Tissue sections were baked at 60°C for 60 min and then placed in Leica Bond III for staining. The procedure is as follows: BOND Dewax, BOND Epitope Retrieval 2 Antigen repair at 97°C for 20 min, endogenous peroxidase blocking for 10 min, primary antibody incubation for 15 min, BOND Polymer Refine Detection for secondary antibody incubation for 16 min. DAB color development for 10 min, hematoxylin redyeing for 2 min after returning to blue. Sy: Clone DAK‐SYNAP, ready‐to‐use antibody, supplier Dako, staining platform Leica Bond III. CgA: Clone 317F1D8, ready‐to‐use antibody, supplier Suzhou Baidao, staining platform Leica Bond III. CD56: Clone MX039, dilution ratio 1:600, supplier Fuzhou Maixin, dyeing platform Leica Bond III.

### 2.4 Statistical analysis

Statistical analysis was conducted using SPSS 26.0 (IBM Corp, Armonk, NY, USA) and GraphPad Prism 9 (GraphPad Software, San Diego, California USA). Counting data were expressed using frequency and percentage, while measurement data were represented by median and quartile. Categorical variables were compared using the chi‐squared test or Fisher's exact test, and continuous variables were compared using the *t*‐test or Mann–Whitney *U* test. To mitigate potential confounding factors among different pathological types, Propensity Score Matching (PSM) was implemented. The survival rate was evaluated using the Kaplan–Meier method, and the log‐rank test was used for survival rate comparisons. Cox proportional hazards regression analysis was performed to calculate hazard ratios (HRs) with 95% confidence interval (CI) using cancer‐related death as the endpoint. In the Cox regression multivariate analysis, variables with *p*‐values <0.05 were considered significant for inclusion, while variables with *p*‐values >0.1 were considered significant for removal. HRs and their 95% CI were determined for each key variable and compared to their reference category. A *p*‐value <0.05 was considered statistically significant.

## 3 RESULTS

### 3.1 General clinicopathological characteristics

A total of 142 cases of g‐NENs were included in the study. These consisted of 23 cases of NET‐G1 (16.2%), eight cases of NET‐G2 (5.6%), 83 cases of g‐NEC (58.5%), and 28 cases of g‐MiNEN (19.7%). No cases of NET‐G3 were observed. The majority of patients were male (68.3%) with a median age of 63 years. Most tumors were located in the upper third of the stomach (41.5%). 40.1% of patients with g‐NENs experienced recurrence or metastasis, with 24.6% of cases presenting metastasis at initial diagnosis. Distant lymph node and liver metastases were the most common (29.8% and 47.4%, respectively). HER2 expression was positive in only 6.3% of cases, all of which were g‐MiNEN. Detailed clinical and pathological characteristics are presented in Table 1.

### 3.2 g‐NEC/MiNEN is more prevalent in older males and exhibits a higher propensity for recurrence or metastasis compared to g‐NET

**FIGURE 2:**
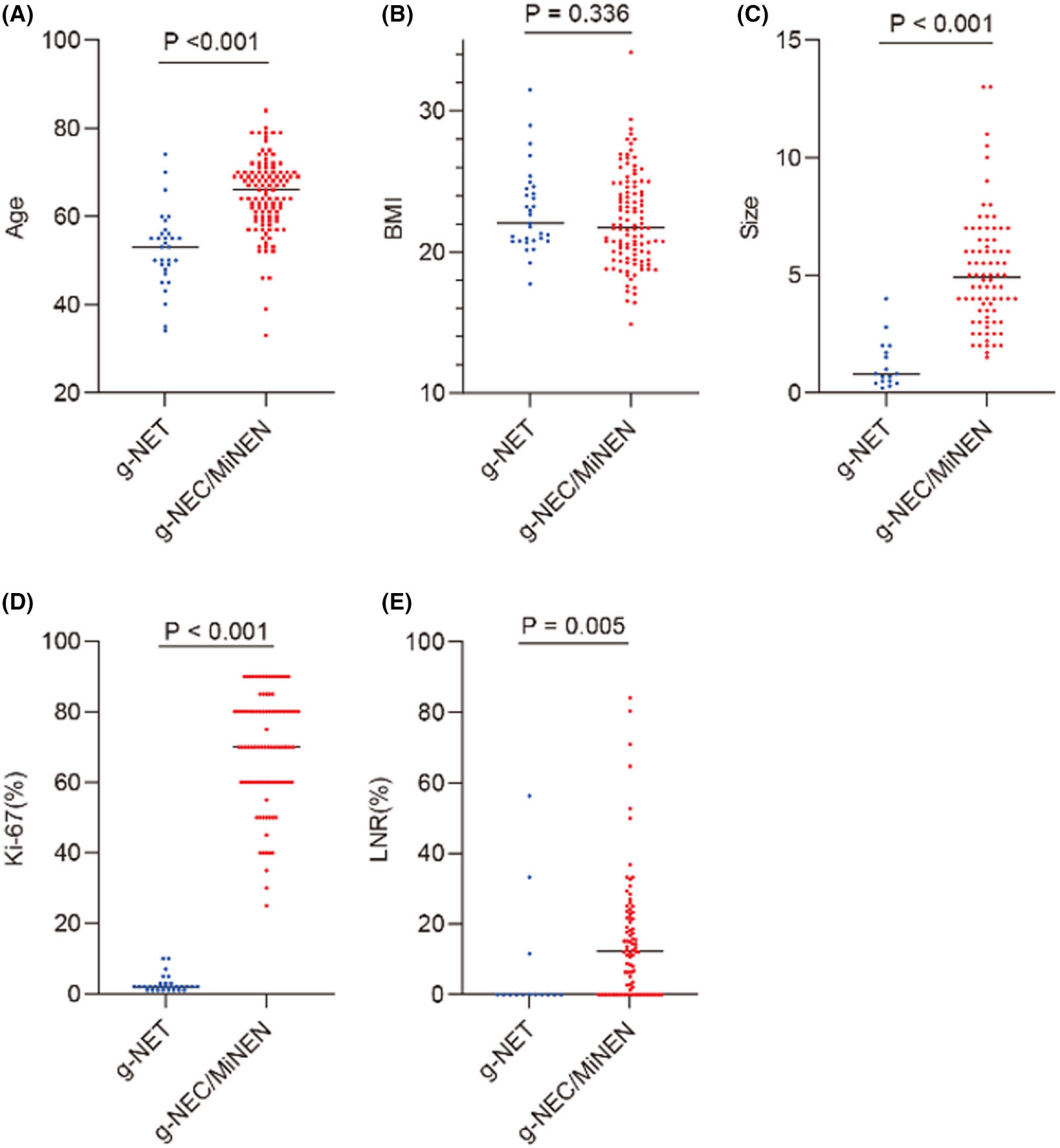
Diagram of the *T*‐test or Mann–Whitney *U* test for differences between groups of continuous variables for g‐NET and g‐NEC/MiNEN. (A) The age of g‐NEC/MiNEN group was significantly higher than that of g‐NET group (*p* < 0.001); (B) There was no difference in BMI between g‐NET and g‐NEC/MiNEN (*p* = 0.336). (C) g‐NEC/MiNEN were significantly larger than g‐NET (*p* < 0.001); (D) The Ki‐67 (%) index of g‐NEC/MiNEN was significantly higher than that of g‐NET (*p* < 0.001); (E) The LNR (%) of g‐NEC/MiNEN is higher than g‐NET (*p* = 0.005).

Compared analysis between G1 and G2 showed no significant differences in clinicopathological characteristics and overall survival (OS) (*p* = 0.065, Table S2 and Figure S1). Similarly, a comparative analysis between g‐MiNEN and g‐NEC groups displayed no significant disparities between them. However, g‐NEC cases demonstrated a higher probability of distant metastasis (*p* = 0.019), a noticeable reduction in BMI (*p* = 0.016), and a lower rate of HER2 positivity (*p* < 0.001, Table S3 and Figure S2). Given the absence of significant differences in clinicopathological features between G1 and G2, as well as g‐MiNEN and g‐NEC, and considering the lack of statistically significant differences in OS, we combined G1 and G2 into the g‐NET group, and grouped g‐MiNEN and g‐NEC as the g‐NEC/MiNEN group. Subsequently, a comparative analysis was performed between these two groups. Notably, male patients had a significantly higher prevalence of g‐NEC/MiNEN (*p* < 0.001) and were older in age (*p* < 0.001). Additionally, the g‐NEC/MiNEN subgroup exhibited a higher likelihood of experiencing recurrence or metastasis (*p* = 0.024), often presenting with distant metastasis at the time of initial diagnosis (*p* = 0.008), as outlined in Table 2 and illustrated in Figure 2.

**TABLE 2:** Chi‐square tests were conducted for g‐NET and g‐NEC/MiNEN groups.

Variable	g‐NET (*n* = 31)	g‐NEC/MiNEN (*n* = 111)	X^2^	*p*
Gender				
Male	8	89	33.095	**<0.001**
Female	23	22		
Smoking history				
No	27	57	12.815	**<0.001**
Yes	4	54		
Drinking history				
No	24	69	2.496	0.114
Yes	7	42		
Family history				
No	22	81	0.049	0.825
Yes	9	30		
Weight loss				
No	28	65	10.818	**0.001**
Yes	3	46		
Tumor location				
Upper 1/3 of the stomach	5	54	21.367	**<0.001**
Middle 1/3 of the stomach	22	29		
Lower 1/3 of the stomach	4	28		
Distant metastasis in initial diagnosis				
No	29	78	7.070	**0.008**
Yes	2	33		
Recurrence or metastasis				
No	24	61	5.089	**0.024**
Yes	7	50		
Recurrence or metastasis sites				
Liver	2	25	0.435	0.510
Other	5	25		
Surgery				
No	1	26	6.420	**0.011**
Yes	30	85		
Objective of surgery				
Radical	29	83	–	1.000
Palliative	1	2		
Method of surgery				
Open	7	74	–	–
Laparoscope	7	11		
ESD	16	0		
Range of surgery				
Whole stomach	11	62	12.585	**<0.001**
Partial stomach	19	23		
Neoadjuvant therapy				
No	30	67	–	–
Yes	0	18		
Postoperative adjuvant therapy				
No	29	32	31.010	**<0.001**
Yes	1	53		
Nerve invasion				
No	14	42	–	–
Yes	0	43		
Vascular tumor thrombus				
No	10	30	6.518	**0.011**
Yes	4	55		
T stage				
1 + 2	15	15	33.337	**<0.001**
3 + 4	1	69		
N stage				
0	27	22	36.725	**<0.001**
1 + 2 + 3	3	62		
M stage				
0	29	85	4.409	**0.036**
1	2	26		
TNM stage				
I + II	13	26	17.722	**<0.001**
III + IV	4	78		
LNR (%)				
≤11.65	12	39	7.635	**0.006**
>11.65	2	46		
CgA				
Negative	0	30	–	–
Positive	29	78		
CD56				
Negative	2	21	3.408	0.065
Positive	26	71		
HER2				
Negative	5	81	–	–
Positive	0	9		

*P* < 0.05 are in bold.

### 3.3 Prognostic factors for g‐NENs

#### 3.3.1 g‐NEC/MiNEN as a risk factor

**FIGURE 3:**
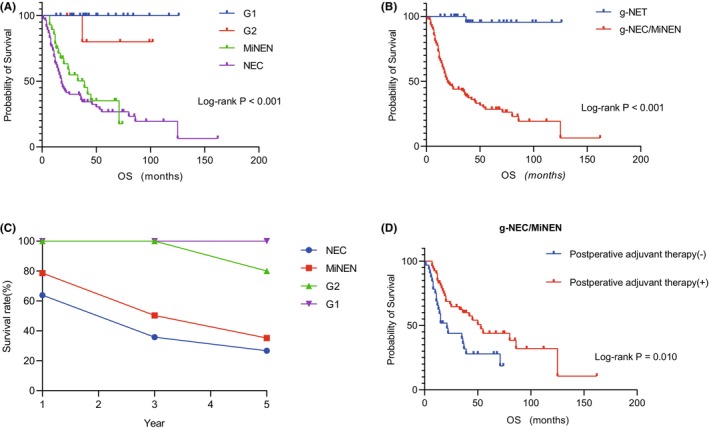
Kaplan–Meier survival curve and survival line chart. (A) Kaplan–Meier survival curves of g‐NENs with four different pathological types, G1, G1, MiNEN, and NEC; (B) Kaplan–Meier survival curves for g‐NET and g‐NEC/MiNEN showed poor prognosis for g‐NEC/MiNEN (*p* < 0.001); (C) 1‐, 3‐, and 5‐year survival rates of g‐NENs with four different pathologic types, G1, G1, MiNEN, and NEC. The 5‐year survival rate of G1 was 100%. The 1‐year, 3‐year, and 5‐year survival rates of G2 were 100.0%, 100.0%, and 80.0%, respectively. The 1‐year, 3‐year, and 5‐year survival rates of MiNEN were 78.6%, 50.3%, and 35.2%, respectively. The 1‐year, 3‐year, and 5‐year survival rates of NEC were 63.9%, 35.8%, and 26.7%, respectively. (D) Kaplan–Meier survival curve of g‐NEC/MiNEN with or without postoperative adjuvant therapy showed that g‐NEC/MiNEN with postoperative adjuvant therapy had a good prognosis (*p* = 0.010).

To evaluate the impact of different pathological types on the prognosis of g‐NENs, Kaplan–Meier survival curves were constructed (Figure 3A,B). The analysis revealed that g‐NEC/MiNEN had a significantly poorer prognosis compared to g‐NET (*p* < 0.001). As illustrated in Figure 3C, the 1‐year, 3‐year, and 5‐year survival rates for g‐NEC were 63.9%, 35.8%, and 26.7%, respectively, with a median survival time of 18 months. For MiNEN patients, the corresponding rates were 78.6%, 50.3%, and 35.2%, with a median survival time of 39 months. G1 patients exhibited a 100% five‐year survival rate, while G2 patients had rates of 100%, 100%, and 80.0% for the 1‐year, 3‐year, and 5‐year periods, respectively.

In order to identify independent prognostic factors for g‐NENs, univariate COX regression analysis was conducted, including clinicopathological data such as age, sex, and TNM stage. The analysis indicated that gender (*p* = 0.002), age (*p* = 0.002), smoking history (*p* = 0.002), pathological type (*p* < 0.001), vascular tumor thrombus (*p* = 0.014), T stage (*p* = 0.003), N stage (*p* < 0.001), M stage (*p* < 0.001), and TNM stage (*p* < 0.001) significantly influenced prognosis (Table S4). Multivariate COX regression analysis was then performed for these factors, but no statistical differences were found among them (Table S5). However, after excluding the tumor stage factor, pathological type (*p* = 0.046) was identified as an independent prognostic factor for g‐NENs (Table S6). These results highlight the potential confounding effect of pathological subtypes on overall prognostic analysis and the identification of independent prognostic factors. Therefore, conducting separate investigations for different pathological subtypes is crucial.

#### 3.3.2 Tumor location, tumor stage, and CD56 expression affect the OS of g‐NET patients

Among the 31 g‐NET patients, two G2 patients initially presented with liver metastasis. One of them showed tumor invasion to the mucosal layer, while the other exhibited invasion to the serous layer. During follow‐up, the patient with serous layer invasion unfortunately succumbed to their condition, while the patient with mucosal layer invasion remained alive until the end of follow‐up. The results from the log‐rank test indicated that tumor location (*p* = 0.042), T stage (*p* = 0.002), N stage (*p* = 0.002), M stage (*p* < 0.001), TNM stage (*p* = 0.034), and CD56 expression level (*p* < 0.001) were significantly associated with the prognosis of gastric neuroendocrine tumors, as detailed in Table 3. Kaplan–Meier survival curves (Figure S3A–F) were generated for each variable, highlighting that lower 1/3 stomach tumors, T3 + T4 stages, lymph node metastasis, distant metastasis, and advanced stages were all correlated with poor prognosis. Additionally, negative CD56 expression was also indicative of an unfavorable prognosis.

**TABLE 3:** Log‐rank test of 31 g‐NET patients.

Variable	g‐NET (n = 31)	X^2^	P
Gender			
Male	8	0.294	0.588
Female	23		
Age(year)			
≤50	14	0.833	0.361
>50	17		
Smoking history			
No	27	0.048	0.827
Yes	4		
Drinking history			
No	24	0.294	0.588
Yes	7		
Family history			
No	22	0.467	0.495
Yes	9		
Weight loss			
No	28	0.100	0.752
Yes	3		
BMI			
<18.5	1	0.467	0.792
18.5–24.0	21		
>24.0	9		
Tumor location			
Upper 1/3 of the stomach	5	6.333	**0.042**
Middle 1/3 of the stomach	22		
Lower 1/3 of the stomach	4		
Tumor size(cm)			
≤1	11	2.000	0.157
>1	6		
Recurrence or metastasis			
No	24	2.667	0.102
Yes	7		
Method of surgery			
Open	7	2.143	0.343
Laparoscope	7		
ESD	16		
Range of surgery			
Whole stomach	11	0.571	0.450
Partial stomach	19		
Nerve invasion			
No	14	–	–
Yes	0		
Vascular tumor thrombus			
No	10	2.333	0.127
Yes	4		
T stage			
1 + 2	15	10.000	**0.002**
3 + 4	1		
N stage			
0	27	10.000	**0.002**
1 + 2 + 3	3		
M stage			
0	29	21.000	**<0.001**
1	2		
TNM stage			
I + II	13	4.500	**0.034**
III + IV	4		
CgA			
Negative	0	–	–
Positive	29		
CD56			
Negative	2	19.000	**<0.001**
Positive	26		
HER2			
Negative	5	–	–
Positive	0		

*P* < 0.05 are in bold.

#### 3.3.3 Postoperative adjuvant therapy prolonged the OS of g‐NEC/MiNEN patients

We constructed a Kaplan–Meier survival curve (Figure 3D) which demonstrated that g‐NEC/MiNEN patients who underwent postoperative adjuvant therapy experienced a significantly prolonged OS (*p* = 0.010). To determine whether postoperative adjuvant therapy serves as an independent prognostic factor for g‐NEC/MiNEN patients, we incorporated gender, age, and TNM stage as clinicopathological characteristics into univariate COX regression analysis. The results of the analysis revealed that postoperative adjuvant therapy (*p* = 0.013), M stage (*p* < 0.001), TNM stage (*p* = 0.015), and lymph node ratio (LNR) (*p* = 0.016) were all associated with the OS of g‐NEC/MiNEN patients. Following this, we included these influencing factors in a multivariate COX regression analysis, and it was found that postoperative adjuvant therapy remained an independent prognostic factor for g‐NEC/MiNEN patients (Table 4). We further performed univariate and multivariate COX analysis on 83 patients with g‐NEC/MiNEN who underwent surgery, and the same result was obtained, that postoperative adjuvant therapy was an independent protective factor for prognosis (Table S7). In light of these findings, our study suggested that both g‐NEC and g‐MiNEN patients may derive benefits from postoperative adjuvant therapy. Therefore, active considering and implementing adjuvant treatment after surgery is recommended to enhance the prognosis of these patients.

**TABLE 4:** Univariate and multivariate COX regression analysis of 111 g‐NEC/MiNEN patients.

g‐NEC/MiNEN	Univariate COX HR(95%CI)	*p*	Multivariate COX HR (95% CI)	*p*
Gender				
Male	1 (Reference)	0.729		
Female	0.904 (0.513, 1.595)			
Age(year)				
≤60	1 (Reference)	0.879		
>60	0.962 (0.583, 1.587)			
Smoking history				
No	1 (Reference)	0.191		
Yes	1.348 (0.861, 2.111)			
Drinking history				
No	1 (Reference)	0.978		
Yes	0.993 (0.626, 1.576)			
Family history				
No	1 (Reference)	0.866		
Yes	0.958 (0.580, 1.582)			
Weight loss				
No	1 (Reference)	0.358		
Yes	1.239 (0.785, 1.956)			
BMI				
<18.5	1 (Reference)	0.533		
18.5–24.0	1.570 (0.687, 3.589)	0.285		
>24.0	1.617 (0.675, 3.871)	0.281		
Tumor location				
Upper 1/3 of the stomach	1 (Reference)	0.784		
Middle 1/3 of the stomach	1.050 (0.600, 1.840)	0.864		
Lower 1/3 of the stomach	1.207 (0.709, 2.053)	0.488		
Tumor size(cm)				
≤5	1 (Reference)	0.760		
>5	1.091 (0.624, 1.907)			
Neoadjuvant therapy				
No	1 (Reference)	0.989		
Yes	0.995 (0.496, 1.996)			
Postoperative adjuvant therapy				
No	1 (Reference)	**0.013**		**0.013**
Yes	0.481 (0.270, 0.855)			0.479 (0.269,0.855)
Range of surgery				
Whole stomach	1 (Reference)	0.241		
Partial stomach	1.426 (0.788, 2.582)			
Nerve invasion				
No	1 (Reference)	0.390		
Yes	1.275 (0.733, 2.220)			
Vascular tumor thrombus				
No	1 (Reference)	0.129		
Yes	1.615 (0.870, 2.997)			
T stage				
1 + 2	1 (Reference)	0.299		
3 + 4	1.531 (0.685, 3.419)			
N stage				
0	1 (Reference)	0.145		
1 + 2 + 3	1.715 (0.830, 3.545)			
M stage				
0	1 (Reference)	**<0.001**		0.416
1	3.393 (2.026, 5.685)		1.547 (0.540, 4.430)	
TNM stage				
I + II	1 (Reference)	**0.015**		0.708
III + IV	2.177 (1.164, 4.072)		1.161 (0.531, 2.541)	
LNR (%)				
≤11.65	1 (Reference)	**0.016**		0.086
>11.65	2.061 (1.147, 3.704)		1.837 (0.918, 3.676)	
CgA				
Negative	1 (Reference)	0.289		
Positive	0.764 (0.464, 1.257)			
CD56				
Negative	1 (Reference)	0.349		
Positive	1.362 (0.714, 2.598)			
HER2				
Negative	1 (Reference)	0.294		
Positive	0.612 (0.245, 1.531)			
Ki‐67(%)				
20–65	1 (Reference)	0.535		
>65	0.852 (0.514, 1.412)			

*P* < 0.05 are in bold.

### 3.4 g‐NEC/MiNEN has poor prognosis compared to GAC and even poorly differentiated GAC

**FIGURE 4:**
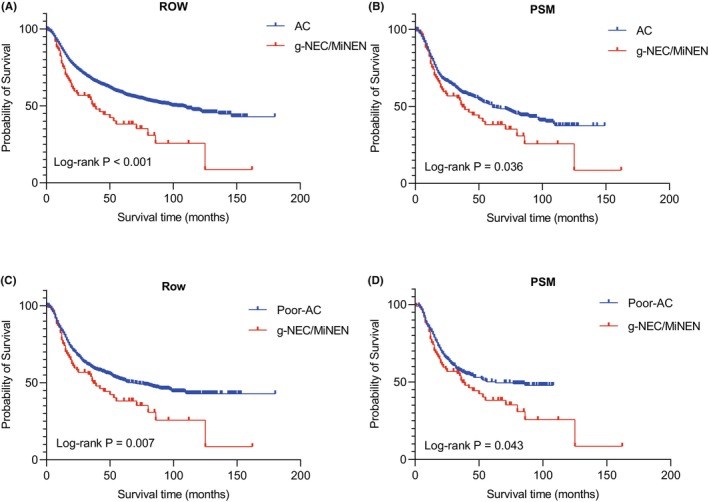
Kaplan–Meier survival curves of gastric adenocarcinoma and poorly differentiated gastric adenocarcinoma before and after PSM and g‐NEC/MiNEN. (A) Kaplan–Meier survival curves of gastric adenocarcinoma and g‐NEC/MiNEN before PSM, with poor prognosis for g‐NEC/MiNEN (*p* < 0.001); (B) Kaplan–Meier survival curves of gastric adenocarcinoma and g‐NEC/MiNEN after PSM showed poor prognosis in g‐NEC/MiNEN (*p* = 0.036). (C) Kaplan–Meier survival curves of poorly differentiated gastric adenocarcinoma and g‐NEC/MiNEN before PSM showed poor prognosis in g‐NEC/MiNEN (*p* = 0.007); (D) Kaplan–Meier survival curves of poorly differentiated gastric adenocarcinoma and g‐NEC/MiNEN after PSM showed poor prognosis in g‐NEC/MiNEN (*p* = 0.043).

GAC is the most common subtype of gastric malignancies. To provide a more comprehensive understanding of g‐NEC/MiNEN, we incorporated data from 3217 cases of GAC diagnosed within the same time frame at our research center. Our analysis revealed several significant differences between g‐NEC/MiNEN and GAC. Notably, the age of onset for g‐NEC/MiNEN was considerably higher than that of GAC (*p* = 0.007). Furthermore, g‐NEC/MiNEN cases were more frequently associated with advanced T stage (T3 + T4, *p* = 0.011), a high incidence of distant metastasis (*p* = 0.013), and a greater proportion of cases in stage III + IV (*p* = 0.018). The 5‐year survival rates for GAC and g‐NEC/MiNEN were 58.4% and 38.2%, respectively, indicating a significantly poorer prognosis for g‐NEC/MiNEN (*p* < 0.001, Table 5 and Figure 4A). In order to eliminate the influence of confounding factors on prognosis, we performed PSM at a ratio of 1:5 according to age, sex, and TNM stage, and finally obtained a balanced cohort of 425 cases of gastric adenocarcinoma and 85 cases of g‐NEC/MiNEN. The postmatching analysis confirmed that g‐NEC/MiNEN continued to exhibit a poor prognosis (*p* = 0.036, Figure 4B).

**TABLE 5:** Chi‐square test before and after PSM gastric adenocarcinoma and g‐NEC/MiNEN.

Pre‐PSM	GAC (*n* = 3217)	g‐NEC/MiNEN (*n* = 85)	X^2^	*p*	Post‐PSM GAC (*n* = 425)	g‐NEC/MiNEN (*n* = 85)	X^2^	*p*
Variable								
Gender								
Male	2460	68	0.575	0.448	340	68	0	1.000
Female	757	17			68	17		
Age(year)								
≤60	1258	21	7.235	**0.007**	105	21	0	1.000
>60	1959	64			320	64		
BMI								
<18.5	314	8	2.819	0.244	47	8	2.492	0.288
18.5–24.0	2053	47			267	47		
>24.0	850	29			111	29		
Family history								
No	2150	63	1.988	0.159	298	63	0.548	0.459
Yes	1067	22			127	22		
Smoking history								
No	1788	46	0.072	0.789	238	46	0.102	0.750
Yes	1429	39			187	39		
Drinking history								
No	2231	52	2.593	0.107	311	52	4.972	**0.026**
Yes	986	33			114	33		
Tumor location								
Upper 1/3 of the stomach	904	47	40.897	**<0.001**	132	47	27.691	**<0.001**
Middle 1/3 of the stomach	508	19			69	19		
Lower 1/3 of the stomach	1726	19			211	19		
Whole stomach	79	0			13	0		
Vascular tumor thrombus								
No	1638	30	8.119	**0.004**	197	30	3.507	0.061
Yes	1577	55			228	55		
Nerve invasion								
No	1666	42	0.192	0.661	200	42	0.157	0.692
Yes	1549	43			225	43		
Tumor size(cm)								
≤5	2099	47	3.065	0.080	252	47	0.277	0.599
>5	1054	35			165	35		
T stage								
1 + 2	989	15	6.422	**0.011**	95	15	0.837	0.360
3 + 4	2228	69			330	69		
N stage								
0	1124	23	2.269	0.132	107	23	0.132	0.716
1 + 2 + 3	2093	62			318	62		
M stage								
0	3133	79	6.179	**0.013**	413	79	3.732	0.053
1	84	6			12	6		
TNM stage								
I + II	1412	26	5.575	**0.018**	130	26	0.004	0.947
III + IV	1805	58			295	58		
HER2								
Negative	2504	64	0.716	0.398	340	64	1.156	0.282
Positive	227	8			27	8		

*P* < 0.05 are in bold.

Given the poor differentiation of g‐NEC/MiNEN, we conducted a separate analysis involving 1007 cases of poor differentiation GAC for comparison. Our findings indicated that male patients were more commonly affected by g‐NEC/MiNEN (*p* = 0.034), and the age of onset for g‐NEC/MiNEN was typically higher than 60 years (*p* < 0.001) when compared to poorly differentiated GAC. Importantly, the prognosis for g‐NEC/MiNEN remained poor (*p* = 0.007), with a 5‐year survival rate lower than that of poorly differentiated GAC, reaching 51.5% (Figure 4C, Table S8). To address potential confounding factors, we performed PSM at a ratio of 1:5 based on age, sex, and TNM stage. The analysis following PSM demonstrated that g‐NEC/MiNEN continued to have a poor prognosis compared to poorly differentiated GAC (*p* = 0.043, Figure 4D).

SRCC is a distinct subtype of gastric adenocarcinoma. To explore potential differences in prognosis between g‐NEC/MiNEN and SRCC, we conducted an in‐depth analysis and comparison of clinicopathological characteristics and OS using data from 255 cases of gastric SRCC from the same research center during the same period. The analysis demonstrated that g‐NEC/MiNEN is associated with a poorer prognosis when compared to SRCC, particularly in early cases (Table S9, Figure S4).

## 4 DISCUSSION

The rarity and significant clinicopathological heterogeneity of gastric neuroendocrine neoplasms (g‐NENs) pose a formidable challenge for clinicians in terms of prognostication and treatment selection. It is widely recognized that tumor location and histological characteristics play a pivotal role in determining the biological behavior and prognosis of neuroendocrine neoplasms.^14^, ^15^, ^16^, ^17^ The latest classification by the WHO for digestive system tumors stratifies g‐NENs into distinct categories: well‐differentiated g‐NET, poorly differentiated g‐NEC and g‐MiNEN.^8^ In our study, the distribution across these subtypes revealed a total of 31 cases of g‐NET (21.8%), 83 cases of g‐NEC (58.5%), and 28 cases of g‐MiNEN (19.7%). Remarkably, g‐NEC constituted more than half of these cases, diverging from the existing epidemiological data, where g‐NETs tend to predominate.^5^, ^9^, ^18^, ^19^, ^20^, ^21^, ^22^ This discrepancy might stem from the fact that our hospital specializes in the treatment of malignant tumors, potentially introducing a selection bias.

To gain further insights into the clinicopathological characteristics of g‐NENs, we conducted an intergroup analysis for each pathological subtype. Surprisingly, we found no discernible differences in fundamental clinicopathological features and prognosis between the G1 and G2 groups. The lack of statistical significance in the comparison of G1 and G2 may be simply due to the small sample size of the two groups. However, G1 and G2 had a good prognosis and were combined into one group for analysis, which did not affect our study of the final results. Similarly, when performing an intergroup analysis for g‐NEC and g‐MiNEN, we identified no significant disparities in their basic clinicopathological features or prognosis. As a result, we combined the G1 and G2 groups into a unified g‐NET category, while amalgamating the g‐NEC and g‐MiNEN groups to establish a consolidated g‐NEC/MiNEN category. A comparison between the g‐NET group and the g‐NEC/MiNEN group highlighted a higher prevalence of the latter among elderly males, consistent with findings from numerous studies.^23^, ^24^, ^25^, ^26^, ^27^, ^28^, ^29^, ^30^, ^31^ Additionally, we noted a heightened prevalence of smoking history within this patient population, potentially with a gender‐related association. Our study unveiled that g‐NEC/MiNEN predominantly manifested in the upper third of the stomach, aligning with Liu et al.'s discovery that g‐NEC primarily occurs in the cardia region.^27^ Similarly, a higher incidence of relapses or metastases was observed in the g‐NEC/MiNEN group compared to the g‐NET group. Furthermore, a larger number of patients presented with distant metastases during their initial visit, primarily affecting the liver and lymph nodes. These findings consistently concur with previous investigations by multiple researchers.^12^, ^27^, ^32^, ^33^, ^34^, ^35^, ^36^, ^37^ In the g‐NET group, no cases tested positive for HER2, while in the g‐NEC/MiNEN group, only nine cases were HER2 positive, all of which were g‐MiNEN. As far back as 2014, Ishida et al. examined HER2 expression in 51 g‐NEC cases and found a lack of HER2 expression in all NEC cases.^38^ In 2021, Yamashita et al. similarly identified negative HER2 expression in all NEC tissue cases, with HER2 positivity observed in 15 (40%) MiNEN adenocarcinoma components.^39^ The HER2 positive rate in g‐NENs is significantly lower than that in GAC, which could be attributed to the nonexpression of HER2 in g‐NETs and the limited expression in g‐NEC/MiNENs. Consequently, HER2 is unlikely to serve as an effective treatment target, diverging from its role in GAC.

The prognosis of g‐NET, g‐NEC, and g‐MiNEN is believed to display significant differences. Specifically, g‐NET demonstrates indolent growth patterns and a propensity towards benign tumor behavior,^40^, ^41^ while both g‐NEC and g‐MiNEN are marked by high malignancy and unfavorable prognostic outcomes.^42^, ^43^, ^44^ Our study unveiled that the 5‐year survival rates for G1 and G2 were 100% and 80.0%, respectively. By contrast, g‐NEC and g‐MiNEN exhibited considerably lower rates of 26.7% and 35.2%, respectively. These findings underscore the adverse prognosis associated with g‐NEC and g‐MiNEN, juxtaposed with the favorable outcomes observed for G1 and G2. Delving deeper into the prognostic factors within the g‐NET group, we discovered that tumor location in the lower third of the stomach, T3 + T4 stage, lymph node and distant metastasis, as well as stage III + IV were all linked to a poorer prognosis. Additionally, patients exhibiting negative CD56 expression experienced unfavorable outcomes. It is well known that CD56 expression is mainly associated with, but not limited to, natural killer cells. It has been reported that CD56 is also expressed in GEP‐NENs,^45^ but the role of CD56 in G‐NET remains unclear. NK cell infiltration has not been reported in G‐NET, but the current research mainly focuses on PD‐1/PD‐L1 in GEP‐NEN. In 2022, Kurtulan et al. conducted a study on g‐NETs and identified grade, size, and depth of lesion infiltration as significant determinants of prognosis.^46^ Other studies have reported diverse prognostic factors. Overall, the TNM stage remains a critical prognostic factor.^47^, ^48^, ^49^, ^50^, ^51^, ^52^, ^53^, ^54^

When investigating the prognostic factors of g‐NEC and g‐MiNEN, we found that postoperative adjuvant therapy can extend the OS of patients with g‐NEC and g‐MiNEN, and it emerges as an independent prognostic factor of g‐NEC. We classified postoperative adjuvant therapy into categories such as adjuvant chemotherapy, adjuvant radiotherapy, and immunotherapy. A majority of patients with g‐NEC and g‐MiNEN underwent surgical treatment alongside comprehensive treatment, including surgery and postoperative adjuvant therapy. Recent years have witnessed surgical resection establishing itself as the most effective therapeutic approach for g‐NEC.^31^, ^55^, ^56^ Drawing from a comprehensive and thorough comprehension of g‐NEC and g‐MiNEN, most researchers agree that postoperative adjuvant chemotherapy could offer potential benefits to these patients.^27^, ^57^, ^58^, ^59^, ^60^ However, it is important to note that certain studies have reported the ineffectiveness of adjuvant chemotherapy in treating g‐NEC and g‐MiNEN, failing to demonstrate significant enhancements in survival outcomes.^61^, ^62^ The variations in treatment outcomes can be attributed to several factors, including tumor heterogeneity, drug selection, and population responsiveness. Although our findings suggested that preoperative neoadjuvant therapy did not exert a substantial impact on OS, a study by Ma et al. demonstrated improved prognosis and survival in patients with locally advanced g‐NEC or g‐MiNEN who received preoperative neoadjuvant therapy.^44^ Other treatment approaches have also exhibited promise, such as targeting PD‐1/PD‐L1 and CTLA‐4.^39^, ^63^ Considering the high invasiveness and poor prognosis associated with g‐NEC and g‐MiNEN, our research suggests that a comprehensive treatment approach should be administered to patients g‐NEC and g‐MiNEN after surgery in order to prolong their survival.

The most prevalent pathological type of gastric cancer (GC) is adenocarcinoma.^64^ However, recent epidemiological data indicate a decline in its incidence, while the incidence of g‐NEC is rapidly increasing.^65^ Our findings indicated that g‐NEC/MiNEN exhibited a worse prognosis even compared to poorly differentiated GAC. This result aligned with conclusions drawn by numerous researchers in the field.^30^, ^32^, ^43^, ^66^, ^67^, ^68^, ^69^, ^70^, ^71^, ^72^, ^73^, ^74^, ^75^, ^76^ In 2006, Jiang et al. found that the 5‐year OS rate for g‐NEC and GAC were 31.1% and 69.3%, respectively, with significant differences in OS observed at each stage between g‐NEC and GAC.^70^ Other researchers suggested that the presence of neuroendocrine components indicated a poor prognosis for GC.^66^, ^75^ However, a study conducted by Li et al. in 2022 yielded different results when comparing the OS and cancer‐specific survival (CSS) rates between these two types. They concluded that g‐NEC in individuals of Caucasian descent exhibited a more favorable prognosis than GAC. The dissimilarity in results between Li et al.'s study and previous research may potentially be attributed to ethnic disparities, given that most reported studies have been based on data from Asian populations. Meanwhile, we conducted a comparison between two groups of SRCC, which is another rare pathological type of GC. Our findings revealed that both pre‐ and post‐operative‐g‐NEC/MiNEN patients had a poorer prognosis than SRCC. Further stratified analysis indicated that g‐NEC/MiNEN in the early stage had a worse prognosis compared to SRCC, but there was no significant difference in the prognosis of advanced g‐NEC/MINEN cases. In conclusion, our study suggested that g‐NEC/MiNEN exhibited higher malignancy and a worse prognosis than SRCC in the early stage, while their malignancy appeared to be similar in the advanced stage.

Our study has a few of limitations. First, this is a retrospective study, which inevitably results in selection bias and information bias during data collection process. Second, the sample size in this single‐center study is relatively small, as it only includes samples from our hospital. To enhance the generalizability and robustness of our findings, future research should involve multiple research centers and incorporate larger sample sizes.

However, our research does offer a few of advantages. First, we included all patients with gastric neuroendocrine neoplasms treated at our hospital over the past 15 years and reclassified them according to the latest WHO classification of digestive system tumors, distinguishing between G1, G2, G3, NEC, and MiNEN subtypes. Subsequently, we conducted a comprehensive analysis of clinicopathological characteristics and prognosis from various perspectives for each pathological type as well as for the entire cohort. Second, we pioneered a comparative analysis between g‐NEC/MiNEN and SRCC, shedding light on the fact that g‐NEC/MiNEN exhibited high malignancy and poor prognosis in the early stages, while its malignancy level becomes similar to that of advanced stage. This unique aspect of our study contributes to the current body of knowledge in this field.

Our study highlights several key findings. g‐NENs tend to manifest in elderly male patients and often the loss of HER2 expression. g‐NETs generally exhibit a favorable prognosis, with the TNM stage emerging as a primary prognostic factor. By contrast, g‐NEC/MiNEN presents a grim prognosis, displaying a heightened likelihood of relapse or metastasis. It frequently presents with distant metastases at the time of initial diagnosis, predominantly affecting the liver and lymph nodes. Postoperative adjuvant therapy has been demonstrated to improve overall survival for g‐NEC and g‐MiNEN cases. Importantly, g‐NEC/MiNEN demonstrates elevated malignancy and worse prognostic outcomes in comparison to GAC, and even poorly differentiated GAC. Notably, early‐stage g‐NEC/MiNEN shows greater malignancy than SRCC and accompanies a poor prognosis. Conversely, advanced‐stage g‐NEC/MINEN shares similar malignant characteristics with SRCC. These insights collectively contribute to our understanding of gastric neuroendocrine neoplasms and have potential implications for treatment strategies and prognostic evaluations.

## AUTHOR CONTRIBUTIONS

**Mengli Zi:** Data curation (equal); investigation (equal); methodology (equal); writing – original draft (equal). **Yubo Ma:** Investigation (equal); methodology (equal); writing – original draft (equal). **Jinxia Chen:** Data curation (equal); methodology (equal). **Chuhong Pang:** Data curation (equal). **xiao Li:** Data curation (equal); methodology (equal). **Li Yuan:** Investigation (equal); methodology (equal); validation (equal). **Zhuo Liu:** Writing – review and editing (equal). **Pengfei Yu:** Conceptualization (equal); writing – review and editing (equal).

## FUNDING INFORMATION

This study was supported by National Key R&D Program of China (2021YFA0910100), National Natural Science Foundation of China (82074245, 81973634, 82204828), Medical Science and Technology Project of Zhejiang Province (2022KY114, WKJ‐ZJ‐2104), Natural Science Foundation of Zhejiang Province (HDMY22H160008), Science and Technology Projects of Zhejiang Province (2019C03049).

## CONFLICT OF INTEREST STATEMENT

The authors have no conflict of interest.

## ETHICS STATEMENT

The study was approved by the Institutional Ethics Committee of Zhejiang Cancer Hospital (no. IRB‐2023‐1005) and informed consent has been waived by the Ethic Committee.

## Supporting information


Appendix S1

## Data Availability

The data that support the findings of this study are available on request from the corresponding author. The data are not publicly available due to privacy or ethical restrictions.
