# Supplementary material for: Clinicopathological characteristics of gastric neuroendocrine neoplasms: A comprehensive analysis
Source: Cancer Med. 2024 Mar 8;13(4):e7011. doi: 10.1002/cam4.7011 (PMC10922030; doi:10.1002/cam4.7011)
Supplement: Supplementary file 1 — Appendix S1 [file CAM4-13-e7011-s001.docx]

**
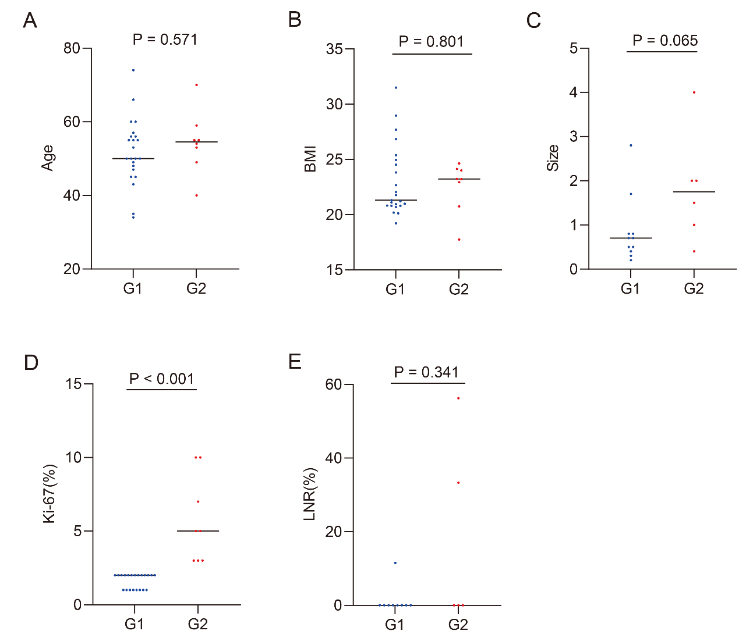
**

**Supplementary Figure 1：**Diagram of T-test or Mann-Whitney U test for differences between continuous variables of G1 and G2. **A**: There was no significant difference in age between G1 and G2 (P=0.571). **B**: There was no significant difference in BMI between G1 and G2 (P=0.801). **C**: There was no significant difference in tumor size between G1 and G2 (P=0.065). **D**: The Ki-67 (%) index of G2 was higher than that of G1 (P<0.001); **E**: There was no significant difference in LNR (%) between G1 and G2 (P=0.341).

**
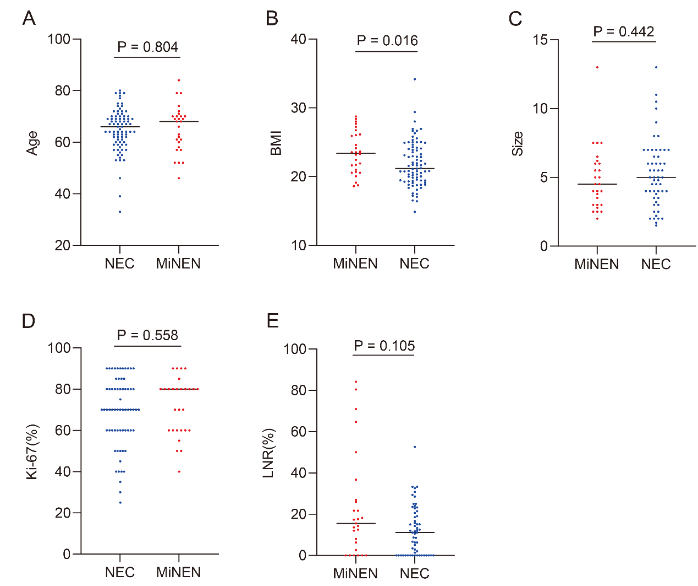
**

**Supplementary Figure 2:** Diagram of the T-test or Mann-Whitney U test for differences between continuous variables of NEC and MiNEN. **A**: There was no significant difference in age between NEC and MiNEN (P=0.804). **B**: The BMI of NEC was significantly lower than that of MiNEN (P=0.016). **C**: There was no significant difference in tumor size between NEC and MiNEN (P=0.442). **D**: There was no significant difference in Ki-67 (%) index between NEC and MiNEN (P=558); **E:** There was no significant difference in LNR (%) between NEC and MiNEN (P=0.105).

**
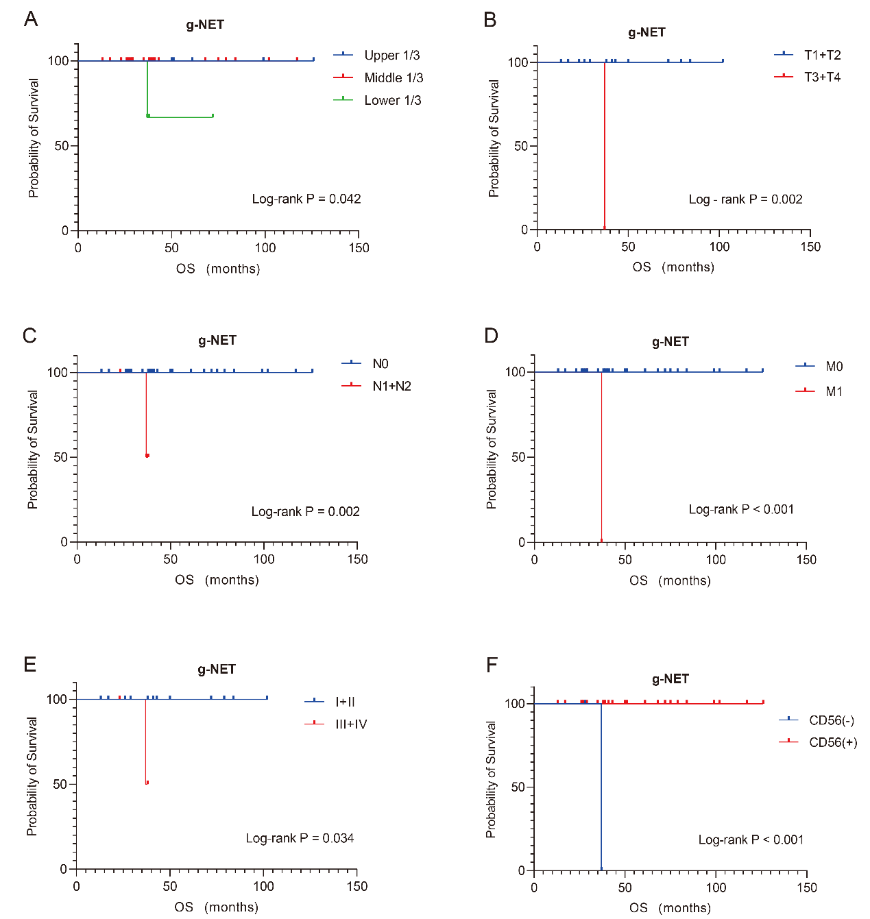
**

**Supplementary Figure 3:** Kaplan-Meier survival curve for g-NET. **A**: g-NET located in the lower 1/3 of the stomach had poor prognosis (P=0.042). **B**: g-NET in T3+T4 stage had poor prognosis (P=0.002); **C**: g-NET with lymph node metastasis had poor prognosis (P=0.002). **D**: g-NET with distant metastasis has a poor prognosis (P<0.001); **E:** g-NET in III+IV stage had poor prognosis (P=0.034); **F**: CD56 negative g-NET had a poor prognosis (P<0.001).

**
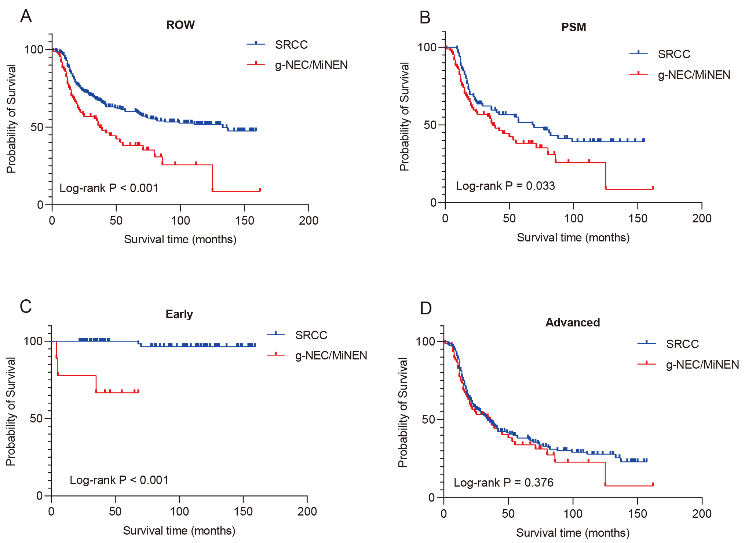
**

**Supplementary Figure** 4: Kaplan-Meier survival curves of PSM anterior and posterior gastric signet ring cell carcinoma and g-NEC/MiNEN. **A**: Kaplan-Meier survival curves of signet-ring cell carcinoma and g-NEC/MiNEN before PSM, with poor prognosis for g-NEC/MiNEN (P<0.001); **B**: Kaplan-Meier survival curves of signet-ring cell carcinoma and g-NEC/MiNEN after PSM showed poor prognosis in g-NEC/MiNEN (P=0.033); **C**: Kaplan-Meier survival curve of early signet ring cell carcinoma and g-NEC/MiNEN showed poor prognosis (P<0.001); D: Kaplan-Meier survival curves of advanced signet ring cell carcinoma and g-NEC/MiNEN showed no difference in prognosis between the two groups (P=0.376).

**Supplementary Table 1** The diagnosis criteria for those 28 cases of MiNEN.

| Case | Types of pathology | Proportion (%) |
| --- | --- | --- |
| 1 | adenocarcinoma | 40 |
|  | neuroendocrine carcinomas | 60 |
| 2 | adenocarcinoma | 60 |
|  | neuroendocrine carcinomas | 40 |
| 3 | adenocarcinoma | 35 |
|  | neuroendocrine carcinomas | 65 |
| 4 | adenocarcinoma | 30 |
|  | neuroendocrine carcinomas | 70 |
| 5 | adenocarcinoma | 60 |
|  | neuroendocrine carcinomas | 40 |
| 6 | adenocarcinoma | 40 |
|  | neuroendocrine carcinomas | 60 |
| 7 | adenocarcinoma | 50 |
|  | neuroendocrine carcinomas | 50 |
| 8 | adenocarcinoma | 60 |
|  | neuroendocrine carcinomas | 40 |
| 9 | adenocarcinoma | 60 |
|  | neuroendocrine carcinomas | 40 |
| 10 | adenocarcinoma | 50 |
|  | neuroendocrine carcinomas | 50 |
| 11 | adenocarcinoma | 30 |
|  | neuroendocrine carcinomas | 70 |
| 12 | adenocarcinoma | 30 |
|  | neuroendocrine carcinomas | 70 |
| 13 | adenocarcinoma | 30 |
|  | neuroendocrine carcinomas | 70 |
| 14 | adenocarcinoma | 50 |
|  | neuroendocrine carcinomas | 50 |
| 15 | adenocarcinoma | 40 |
|  | neuroendocrine carcinomas | 60 |
| 16 | adenocarcinoma | 50 |
|  | neuroendocrine carcinomas | 50 |
| 17 | adenocarcinoma | 40 |
|  | neuroendocrine carcinomas | 60 |
| 18 | adenocarcinoma | 50 |
|  | neuroendocrine carcinomas | 50 |
| 19 | adenocarcinoma | 40 |
|  | neuroendocrine carcinomas | 60 |
| 20 | adenocarcinoma | 50 |
|  | neuroendocrine carcinomas | 50 |
| 21 | adenocarcinoma | 70 |
|  | neuroendocrine carcinomas | 30 |
| 22 | adenocarcinoma | 30 |
|  | neuroendocrine carcinomas | 70 |
| 23 | mucinous adenocarcinoma | 40 |
|  | neuroendocrine carcinomas | 60 |
| 24 | signet ring cell carcinoma | 50 |
|  | neuroendocrine carcinomas | 50 |
| 25 | adenocarcinoma | 40 |
|  | neuroendocrine carcinomas | 60 |
| 26 | adenocarcinoma | 50 |
|  | neuroendocrine carcinomas | 50 |
| 27 | adenocarcinoma | 30 |
|  | neuroendocrine carcinomas | 70 |
| 28 | adenocarcinoma | 40 |
|  | neuroendocrine carcinomas | 60 |

**Supplementary Table 2** Chi-square tests were conducted for G1 and G2 groups.

| Variable | G1  （n=23） | G2  （n=8） | X^2^ | P |
| --- | --- | --- | --- | --- |
| Gender |  |  |  |  |
| Male | 6 | 2 | 0 | 1.000 |
| Female | 17 | 6 |  |  |
| Smoking history |  |  |  |  |
| No | 21 | 6 | 0.328 | 0.567 |
| Yes | 2 | 2 |  |  |
| Drinking history |  |  |  |  |
| No | 19 | 5 | 0.464 | 0.496 |
| Yes | 4 | 3 |  |  |
| Family history |  |  |  |  |
| No | 16 | 6 | 0 | 1.000 |
| Yes | 7 | 2 |  |  |
| Weight loss |  |  |  |  |
| No | 22 | 6 | - | 0.156 |
| Yes | 1 | 2 |  |  |
| Tumor location |  |  |  |  |
| Upper 1/3 of the stomach | 5 | 0 | - | - |
| Middle 1/3 of the stomach | 17 | 5 |  |  |
| Lower 1/3 of the stomach | 1 | 3 |  |  |
| Distant metastasis at first diagnosis |  |  |  |  |
| No | 23 | 6 | - | 0.060 |
| Yes | 0 | 2 |  |  |
| Recurrence or metastasis |  |  |  |  |
| No | 20 | 4 | 2.764 | 0.096 |
| Yes | 3 | 4 |  |  |
| Recurrent or metastatic sites |  |  |  |  |
| Liver | 0 | 2 | - | - |
| Other | 3 | 2 |  |  |
| surgery |  |  |  |  |
| No | 0 | 1 | - | - |
| Yes | 23 | 7 |  |  |
| Objective of surgery |  |  |  |  |
| Radical | 23 | 6 | - | - |
| Palliative | 0 | 1 |  |  |
| Method of surgery |  |  |  |  |
| Open | 4 | 3 | 2.649 | 0.266 |
| Laparoscope | 5 | 2 |  |  |
| ESD | 14 | 2 |  |  |
| Range of surgery |  |  |  |  |
| Whole stomach | 8 | 3 | 0 | 1.000 |
| Partial stomach | 15 | 4 |  |  |
| Neoadjuvant therapy |  |  |  |  |
| No | 23 | 7 | - | - |
| Yes | 0 | 0 |  |  |
| Postoperative adjuvant therapy |  |  |  |  |
| No | 23 | 6 | - | - |
| Yes | 0 | 1 |  |  |
| Nerve invasion |  |  |  |  |
| No | 9 | 5 | - | - |
| Yes | 0 | 0 |  |  |
| Vascular tumor thrombus |  |  |  |  |
| No | 8 | 2 | 1.750 | 0.186 |
| Yes | 1 | 3 |  |  |
| T stage |  |  |  |  |
| 1+2 | 10 | 5 | - |  |
| 3+4 | 0 | 1 |  |  |
| N stage |  |  |  |  |
| 0 | 22 | 5 | - | 0.128 |
| 1+2+3 | 1 | 2 |  |  |
| M stage |  |  |  |  |
| 0 | 23 | 6 | - | - |
| 1 | 0 | 2 |  |  |
| TNM stage |  |  |  |  |
| I+II | 9 | 4 | 0.982 | 0.322 |
| III+IV | 1 | 3 |  |  |
| CgA |  |  |  |  |
| [negative](javascript:;) | 0 | 0 | - | - |
| positive | 21 | 8 |  |  |
| CD56 |  |  |  |  |
| [negative](javascript:;) | 1 | 1 | - | 0.444 |
| positive | 20 | 6 |  |  |
| HER2 |  |  |  |  |
| [negative](javascript:;) | 3 | 2 | - | - |
| positive | 0 | 0 |  |  |

**Supplementary Table 3** Chi-square tests were conducted for MiNEN and NEC groups.

| Variable | MiNEN  （n=28） | NEC  （n=83） | X^2^ | P |
| --- | --- | --- | --- | --- |
| Gender |  |  |  |  |
| Male | 21 | 68 | 0.632 | 0.427 |
| Female | 7 | 15 |  |  |
| Smoking history |  |  |  |  |
| No | 17 | 40 | 1.314 | 0.252 |
| Yes | 11 | 43 |  |  |
| Drinking history |  |  |  |  |
| No | 19 | 50 | 0.516 | 0.472 |
| Yes | 9 | 33 |  |  |
| Family history |  |  |  |  |
| No | 20 | 61 | 0.045 | 0.831 |
| Yes | 8 | 22 |  |  |
| Weight loss |  |  |  |  |
| No | 19 | 46 | 1.334 | 0.248 |
| Yes | 9 | 37 |  |  |
| Tumor location |  |  |  |  |
| Upper 1/3 of the stomach | 12 | 42 | 0.510 | 0.775 |
| Middle 1/3 of the stomach | 8 | 21 |  |  |
| Lower 1/3 of the stomach | 8 | 20 |  |  |
| Distant metastasis at first diagnosis |  |  |  |  |
| No | 23 | 55 | 2.527 | 0.112 |
| Yes | 5 | 28 |  |  |
| Recurrence or metastasis |  |  |  |  |
| No | 18 | 43 | 1.317 | 0.251 |
| Yes | 10 | 40 |  |  |
| Recurrence or metastasis sites |  |  |  |  |
| Liver | 4 | 21 | 0.500 | 0.480 |
| Other | 6 | 19 |  |  |
| surgery |  |  |  |  |
| No | 1 | 25 | 8.227 | **0.004** |
| Yes | 27 | 58 |  |  |
| Objective of surgery |  |  |  |  |
| Radical | 26 | 57 | - | 0.537 |
| Palliative | 1 | 1 |  |  |
| Method of surgery |  |  |  |  |
| Open | 23 | 51 | 0 | 0.997 |
| Laparoscope | 4 | 7 |  |  |
| Range of surgery |  |  |  |  |
| Whole stomach | 20 | 42 | 0.026 | 0.873 |
| Partial stomach | 7 | 16 |  |  |
| Neoadjuvant therapy |  |  |  |  |
| No | 22 | 45 | 0.167 | 0.682 |
| Yes | 5 | 13 |  |  |
| Postoperative adjuvant therapy |  |  |  |  |
| No | 9 | 23 | 0.314 | 0.575 |
| Yes | 18 | 35 |  |  |
| Nerve invasion |  |  |  |  |
| No | 12 | 30 | 0.391 | 0.532 |
| Yes | 15 | 28 |  |  |
| Vascular tumor thrombus |  |  |  |  |
| No | 8 | 22 | 0.556 | 0.456 |
| Yes | 19 | 36 |  |  |
| T stage |  |  |  |  |
| 1+2 | 3 | 12 | 0.650 | 0.420 |
| 3+4 | 24 | 45 |  |  |
| N stage |  |  |  |  |
| 0 | 6 | 16 | 0.324 | 0.569 |
| 1+2+3 | 21 | 41 |  |  |
| M stage |  |  |  |  |
| 0 | 26 | 59 | 5.533 | **0.019** |
| 1 | 2 | 24 |  |  |
| TNM stage |  |  |  |  |
| I+II | 6 | 20 | 0.261 | 0.610 |
| III+IV | 22 | 56 |  |  |
| LNR (%) |  |  |  |  |
| ≤11.65 | 9 | 30 | 2.510 | 0.113 |
| >11.65 | 18 | 28 |  |  |
| CgA |  |  |  |  |
| [negative](javascript:;) | 10 | 20 | 1.187 | 0.276 |
| positive | 18 | 60 |  |  |
| CD56 |  |  |  |  |
| [negative](javascript:;) | 6 | 15 | 0.027 | 0.870 |
| positive | 19 | 52 |  |  |
| Her-2 |  |  |  |  |
| [negative](javascript:;) | 18 | 63 | 14.429 | **<0.001** |
| positive | 8 | 1 |  |  |
| Ki-67 (%) |  |  |  |  |
| 20-65 | 10 | 24 | 0.004 | 0.952 |
| ＞65 | 18 | 42 |  |  |

**Supplementary Table 4** Univariate COX regression analysis of 142 cases of gastric neuroendocrine neoplasms.

| All | Univariate COX | P |
| --- | --- | --- |
|  | HR（95%CI） |  |
| Gender |  |  |
| Male | 1（Reference） | **0.002** |
| Female | 0.413（0.238,0.717） |  |
| Age（year） |  |  |
| ≤60 | 1（Reference） | **0.002** |
| >60 | 2.180（1.334,3.562） |  |
| Smoking history |  |  |
| No | 1（Reference） | **0.002** |
| Yes | 2.045（1.310,3.194） |  |
| Drinking history |  |  |
| No | 1（Reference） | 0.370 |
| Yes | 1.235（0.779,1.958） |  |
| Family history |  |  |
| No | 1（Reference） | 0.754 |
| Yes | 0.923（0.560,1.523） |  |
| BMI |  |  |
| <18.5 | 1（Reference） | 0.863 |
| 18.5-24.0  >24.0 | 1.171（0.517,2.651）  1.261（0.536,2.964） | 0.705  0.595 |
| Tumor size（cm） |  |  |
| ≤4 | 1（Reference） | 0.081 |
| >4 | 1.648（0.939,2.892） |  |
| Neoadjuvant therapy |  |  |
| No | 1（Reference） | 0.199 |
| Yes | 1.577（0.787,3.157） |  |
| Postoperative adjuvant therapy |  |  |
| No | 1（Reference） | 0.224 |
| Yes | 1.409（0.810,2.449） |  |
| Range of surgery |  |  |
| Whole stomach | 1（Reference） | 0.474 |
| Partial stomach | 0.809（0.453,1.445） |  |
| Pathological type |  |  |
| G1+G2 | 1（Reference） | **<0.001** |
| MiNEN + NEC | 34.112（4.740,245.517） |  |
| Nerve invasion |  |  |
| No | 1（Reference） | 0.054 |
| Yes | 1.716（0.991,2.970） |  |
| Vascular tumor thrombus |  |  |
| No | 1（Reference） | **0.014** |
| Yes | 2.171（1.171,4.023） |  |
| T stage |  |  |
| 1+2 | 1（Reference） | **0.003** |
| 3+4 | 3.325（1.493,7.406） |  |
| N stage |  |  |
| 0 | 1（Reference） | **<0.001** |
| 1+2+3 | 4.597（2.234,9.458） |  |
| M stage |  |  |
| 0 | 1（Reference） | **<0.001** |
| 1 | 4.314（2.598,7.163） |  |
| TNM stage |  |  |
| I+II | 1（Reference） | **<0.001** |
| III+IV | 3.312（1.773，6.187） |  |
| CD56 |  |  |
| [negative](javascript:;) | 1（Reference） | 0.646 |
| positive | 0.864（0.465,1.608） |  |
| HER2 |  |  |
| [negative](javascript:;) | 1（Reference） | 0.408 |
| positive | 0.679（0.272,1.698） |  |

**Supplementary Table 5** Multivariate COX regression analysis of 142 cases of gastric neuroendocrine neoplasms 1

| All | Multivariate COX | P |
| --- | --- | --- |
|  | HR（95%CI） |  |
| Gender |  |  |
| Male | 1（Reference） | 0.859 |
| Female | 1.079（0.467,2.493） |  |
| Age（year） |  |  |
| ≤60 | 1（Reference） | 0.204 |
| >60 | 1.579（0.781,3.192） |  |
| Smoking history |  |  |
| No | 1（Reference） | 0.724 |
| Yes | 1.126（0.583,2.176） |  |
| Pathological type |  |  |
| G1+G2 | 1（Reference） | 0.274 |
| MiNEN + NEC | 3.261（0.392,27.123） |  |
| Vascular tumor thrombus |  |  |
| No | 1（Reference） | 0.277 |
| Yes | 1.433（0.749,2.744） |  |
| T stage |  |  |
| 1+2 | 1（Reference） | 0.534 |
| 3+4 | 1.468（0.438,4.913） |  |
| N stage |  |  |
| 0 | 1（Reference） | 0.243 |
| 1+2+3 | 1.994（0.625,6.359） |  |
| M stage |  |  |
| 0 | 1（Reference） | 0.119 |
| 1 | 2.257（0.812,6.274） |  |
| TNM stage |  |  |
| I+II | 1（Reference） | 0.795 |
| III+IV | 0.838（0.220,3.187） |  |

**Supplementary Table 6** Multivariate COX regression analysis of 142 cases of gastric neuroendocrine neoplasms 2

| All | Multivariate COX | P |
| --- | --- | --- |
|  | HR（95%CI） |  |
| Gender |  |  |
| Male | 1（Reference） | 0.864 |
| Female | 1.073（0.479,2.405） |  |
| Age（year） |  |  |
| ≤60 | 1（Reference） | 0.648 |
| >60 | 1.162（0.610,2.211） |  |
| Smoking history |  |  |
| No | 1（Reference） | 0.418 |
| Yes | 1.298（0.691,2.439） |  |
| Pathological type |  |  |
| G1+G2 | 1（Reference） | **0.046** |
| MiNEN + NEC | 8.139（1.039,63.766） |  |
| Vascular tumor thrombus |  |  |
| No | 1（Reference） | 0.082 |
| Yes | 1.738（0.933,3.237） |  |

**Supplementary Table 7** Univariate and multivariate COX regression analysis of 83 g-NEC/MiNEN patients who underwent surgery.

| g-NEC/MiNEN | Univariate COX  HR（95%CI） | P | Multivariate COX  HR（95%CI） | P |
| --- | --- | --- | --- | --- |
| Gender |  |  |  |  |
| Male | 1（Reference） | 0.855 |  |  |
| Female | 0.937（0.465，1.889） |  |  |  |
| Age（year） |  |  |  |  |
| ≤60 | 1（Reference） | 0.607 |  |  |
| >60 | 1.189（0.614,2.305） |  |  |  |
| Smoking history |  |  |  |  |
| No | 1（Reference） | 0.357 |  |  |
| Yes | 1.302（0.743,2.285） |  |  |  |
| Drinking history |  |  |  |  |
| No | 1（Reference） | 0.895 |  |  |
| Yes | 1.039（0.584,1.849） |  |  |  |
| Family history |  |  |  |  |
| No | 1（Reference） | 0.634 |  |  |
| Yes | 0.857（0.454,1.617） |  |  |  |
| Weight loss |  |  |  |  |
| No | 1（Reference） | 0.978 |  |  |
| Yes | 1.008（0.555,1.834） |  |  |  |
| BMI |  |  |  |  |
| <18.5 | 1（Reference） | 0.168 |  |  |
| 18.5-24.0 | 1.707（0.556,5.248） | 0.350 |  |  |
| >24.0 | 2.611（0.826,8.252） | 0.102 |  |  |
| Tumor location |  |  |  |  |
| Upper 1/3 of the stomach | 1（Reference） | 0.584 |  |  |
| Middle 1/3 of the stomach | 0.714（0.333,1.531） | 0.386 |  |  |
| Lower 1/3 of the stomach | 1.107（0.570,2.148） | 0.764 |  |  |
| Tumor size（cm） |  |  |  |  |
| ≤5 | 1（Reference） | 0.784 |  |  |
| >5 | 1.083（0.612,1.915） |  |  |  |
| Neoadjuvant therapy |  |  |  |  |
| No | 1（Reference） | 0.938 |  |  |
| Yes | 1.028（0.511,2.070） |  |  |  |
| Postoperative adjuvant therapy |  |  |  |  |
| No | 1（Reference） | **0.014** | 0.475（0.264,0.856） | **0.013** |
| Yes | 0.477（0.265,0.859） |  |  |  |
| Range of surgery |  |  |  |  |
| Whole stomach | 1（Reference） | 0.282 |  |  |
| Partial stomach | 1.398（0.760,2.572） |  |  |  |
| Nerve invasion |  |  |  |  |
| No | 1（Reference） | 0.385 |  |  |
| Yes | 1.285（0.730,2.262） |  |  |  |
| Vascular tumor thrombus |  |  |  |  |
| No | 1（Reference） | 0.157 |  |  |
| Yes | 1.567（0.841,2.921） |  |  |  |
| T stage |  |  |  |  |
| 1+2 | 1（Reference） | 0.334 |  |  |
| 3+4 | 1.488（0.664,3.332） |  |  |  |
| N stage |  |  |  |  |
| 0 | 1（Reference） | 0.176 |  |  |
| 1+2+3 | 1.655（0.798,3.433） |  |  |  |
| M stage |  |  |  |  |
| 0 | 1（Reference） | 0.535 |  |  |
| 1 | 1.452（0.447,4.716） |  |  |  |
| TNM stage |  |  |  |  |
| I+II | 1（Reference） | 0.184 |  |  |
| III+IV | 1.563（0.809,3.020） |  |  |  |
| LNR (%) |  |  |  |  |
| ≤11.65 | 1（Reference） | **0.023** | 1.995（1.103,3.607） | **0.022** |
| >11.65 | 1.987（1.100,3.592） |  |  |  |
| CgA |  |  |  |  |
| [negative](javascript:;) | 1（Reference） | 0.472 |  |  |
| positive | 0.794（0.423,1.490） |  |  |  |
| CD56 |  |  |  |  |
| [negative](javascript:;) | 1（Reference） | 0.323 |  |  |
| positive | 1.459（0.690,3.086） |  |  |  |
| HER2 |  |  |  |  |
| [negative](javascript:;) | 1（Reference） | 0.399 |  |  |
| positive | 0.641（0.228,1.801） |  |  |  |
| Ki-67（%） |  |  |  |  |
| 20-65 | 1（Reference） | 0.070 |  |  |
| >65 | 0.558（0.297,1.048） |  |  |  |

**Supplementary Table 8** Chi-square test before and after PSM poorly differentiated gastric adenocarcinoma and g-NEC/MiNEN.

| Pre-PSM | Poor-AC  （n=1007） | g-NEC/MiNEN  （n=85） | X^2^ | P | Post-PSM | g-NEC/MiNEN  （n=85） | X^2^ | P |
| --- | --- | --- | --- | --- | --- | --- | --- | --- |
|  |  |  |  |  | Poor-AC  （n=425） |  |  |  |
| Gender |  |  |  |  |  |  |  |  |
| Male | 695 | 68 | 4.492 | **0.034** | 340 | 68 | 0 | 1.000 |
| Female | 312 | 17 |  |  | 85 | 17 |  |  |
| Age（year） |  |  |  |  |  |  |  |  |
| ≤60 | 486 | 21 | 17.487 | **<0.001** | 105 | 21 | 0 | 1.000 |
| >60 | 521 | 64 |  |  | 320 | 64 |  |  |
| BMI |  |  |  |  |  |  |  |  |
| <18.5 | 103 | 8 | 1.957 | 0.376 | 46 | 8 | 1.930 | 0.381 |
| 18.5-24.0 | 628 | 47 |  |  | 264 | 47 |  |  |
| >24.0 | 276 | 29 |  |  | 115 | 29 |  |  |
| Family history |  |  |  |  |  |  |  |  |
| No | 666 | 63 | 2.250 | 0.134 | 299 | 63 | 0.487 | 0.485 |
| Yes | 341 | 22 |  |  | 126 | 22 |  |  |
| Smoking history |  |  |  |  |  |  |  |  |
| No | 594 | 46 | 0.766 | 0.381 | 228 | 46 | 0.006 | 0.937 |
| Yes | 413 | 39 |  |  | 197 | 39 |  |  |
| Drinking history |  |  |  |  |  |  |  |  |
| No | 697 | 52 | 2.351 | 0.125 | 268 | 52 | 0.107 | 0.743 |
| Yes | 310 | 33 |  |  | 157 | 33 |  |  |
| Tumor location |  |  |  |  |  |  |  |  |
| Upper 1/3 of the stomach | 241 | 47 | 45.583 | **<0.001** | 95 | 47 | 44.881 | **<0.001** |
| Middle 1/3 of the stomach | 208 | 19 |  |  | 85 | 19 |  |  |
| Lower 1/3 of the stomach | 556 | 19 |  |  | 244 | 19 |  |  |
| Whole stomach | 2 | 0 |  |  | 1 | 0 |  |  |
| Vascular tumor thrombus |  |  |  |  |  |  |  |  |
| No | 427 | 30 | 1.628 | 0.202 | 166 | 30 | 0.424 | 0.515 |
| Yes | 580 | 55 |  |  | 259 | 55 |  |  |
| Nerve invasion |  |  |  |  |  |  |  |  |
| No | 430 | 42 | 1.438 | 0.230 | 182 | 42 | 1.248 | 0.264 |
| Yes | 5777 | 43 |  |  | 243 | 43 |  |  |
| Tumor size (cm) |  |  |  |  |  |  |  |  |
| ≤5 | 604 | 47 | 0.526 | 0.468 | 250 | 47 | 0.295 | 0.587 |
| >5 | 380 | 35 |  |  | 163 | 35 |  |  |
| T stage |  |  |  |  |  |  |  |  |
| 1+2 | 236 | 15 | 1.362 | 0.243 | 85 | 15 | 0.204 | 0.652 |
| 3+4 | 771 | 69 |  |  | 340 | 69 |  |  |
| N stage |  |  |  |  |  |  |  |  |
| 0 | 276 | 23 | 0.005 | 0.945 | 112 | 23 | 0.018 | 0.893 |
| 1+2+3 | 731 | 62 |  |  | 313 | 62 |  |  |
| M stage |  |  |  |  |  |  |  |  |
| 0 | 971 | 79 | 2.572 | 0.109 | 406 | 79 | 1.018 | 0.313 |
| 1 | 36 | 6 |  |  | 19 | 6 |  |  |
| TNM stage |  |  |  |  |  |  |  |  |
| I+II | 347 | 26 | 0.424 | 0.515 | 130 | 26 | 0.004 | 0.947 |
| III+IV | 660 | 58 |  |  | 295 | 58 |  |  |
| HER2 |  |  |  |  |  |  |  |  |
| [negative](javascript:;) | 845 | 64 | 6.287 | **0.012** | 369 | 64 | 4.070 | **0.044** |
| positive | 35 | 8 |  |  | 17 | 8 |  |  |

**Supplementary Table 9** Chi-square test before and after PSM signet-ring cell carcinoma and g-NEC/MiNEN.

| Pre-PSM | SRCC  （n=255） | g-NEC/MiNEN  （n=85） | X^2^ | P | Post-PSM  SRCC  （n=85） | g-NEC/MiNEN  （n=85） | X^2^ | P |
| --- | --- | --- | --- | --- | --- | --- | --- | --- |
| Variable |  |  |  |  |  |  |  |  |
| Gender |  |  |  |  |  |  |  |  |
| Male | 139 | 68 | 17.393 | **<0.001** | 66 | 68 | 0.141 | 0.707 |
| Female | 116 | 17 |  |  | 19 | 17 |  |  |
| Age（year） |  |  |  |  |  |  |  |  |
| ≤60 | 175 | 21 | 50.370 | **<0.001** | 21 | 21 | 0 | 1.000 |
| >60 | 80 | 64 |  |  | 64 | 64 |  |  |
| BMI |  |  |  |  |  |  |  |  |
| <18.5 | 20 | 8 | 2.956 | 0.228 | 8 | 8 | 4.704 | 0.095 |
| 18.5-24.0 | 169 | 47 |  |  | 60 | 47 |  |  |
| >24.0 | 66 | 29 |  |  | 17 | 29 |  |  |
| Family history |  |  |  |  |  |  |  |  |
| No | 163 | 63 | 2.974 | 0.085 | 52 | 63 | 3.252 | 0.071 |
| Yes | 92 | 22 |  |  | 33 | 22 |  |  |
| Smoking history |  |  |  |  |  |  |  |  |
| No | 164 | 46 | 2.806 | 0.094 | 49 | 46 | 0.215 | 0.643 |
| Yes | 91 | 39 |  |  | 36 | 39 |  |  |
| Drinking history |  |  |  |  |  |  |  |  |
| No | 182 | 52 | 3.089 | 0.079 | 50 | 52 | 0.098 | 0.754 |
| Yes | 73 | 33 |  |  | 35 | 33 |  |  |
| Tumor location |  |  |  |  |  |  |  |  |
| Upper 1/3 of the stomach | 31 | 47 | 75.188 | **<0.001** | 15 | 47 | 33.091 | <0.001 |
| Middle 1/3 of the stomach | 55 | 19 |  |  | 21 | 19 |  |  |
| Lower 1/3 of the stomach | 149 | 19 |  |  | 40 | 19 |  |  |
| Whole stomach | 20 | 0 |  |  | 9 | 0 |  |  |
| Vascular tumor thrombus |  |  |  |  |  |  |  |  |
| No | 179 | 30 | 32.788 | **<0.001** | 54 | 30 | 13.555 | <0.001 |
| Yes | 76 | 55 |  |  | 31 | 55 |  |  |
| Nerve invasion |  |  |  |  |  |  |  |  |
| No | 120 | 42 | 0.141 | 0.707 | 26 | 42 | 6.275 | 0.012 |
| Yes | 135 | 43 |  |  | 59 | 43 |  |  |
| Tumor size (cm) |  |  |  |  |  |  |  |  |
| ≤5 | 148 | 47 | 0.285 | 0.594 | 43 | 47 | 0.394 | 0.530 |
| >5 | 96 | 35 |  |  | 39 | 35 |  |  |
| T stage |  |  |  |  |  |  |  |  |
| 1+2 | 103 | 15 | 14.140 | **<0.001** | 23 | 15 | 2.052 | 0.152 |
| 3+4 | 152 | 69 |  |  | 62 | 69 |  |  |
| N stage |  |  |  |  |  |  |  |  |
| 0 | 103 | 23 | 4.859 | **0.028** | 23 | 23 | 0 | 1.000 |
| 1+2+3 | 152 | 62 |  |  | 62 | 62 |  |  |
| M stage |  |  |  |  |  |  |  |  |
| 0 | 251 | 79 | 4.945 | **0.026** | 83 | 79 | 1.181 | 0.277 |
| 1 | 4 | 6 |  |  | 2 | 6 |  |  |
| TNM stage |  |  |  |  |  |  |  |  |
| I+II | 117 | 26 | 5.775 | **0.016** | 28 | 26 | 0.077 | 0.782 |
| III+IV | 138 | 58 |  |  | 57 | 58 |  |  |
| HER2 |  |  |  |  |  |  |  |  |
| [negative](javascript:;) | 196 | 64 | 10.177 | **0.001** | 63 | 64 | 3.573 |  |
| positive | 3 | 8 |  |  | 1 | 8 |  | 0.059 |
